# Dantrolene-Responsive Muscle Stiffness in a Patient With a Normal Neurologic Exam and EMG: A Case Report

**DOI:** 10.1155/crnm/6647563

**Published:** 2025-11-27

**Authors:** Adam S. Deardorff, Matthew J. Burford, Mark M. Rich

**Affiliations:** ^1^Department of Neuroscience, Cell Biology, and Physiology, Wright State University Boonshoft School of Medicine, Dayton, Ohio, USA; ^2^Department of Clinical Neurosciences, Wright State University Boonshoft School of Medicine, Dayton, Ohio, USA; ^3^Department of Neurology, The Ohio State University Wexner Medical Center, Columbus, Ohio, USA

**Keywords:** aging, calcium, case report, dantrolene, stiffness

## Abstract

Muscle stiffness or rigidity is a common problem yet is addressed in few studies. Patients with muscle rigidity/spasticity due to injury of upper motor neurons or genetic muscle diseases are sometimes treated with dantrolene. It is not widely used to treat muscle tightness in patients with a negative workup. Here, we present a 62-year-old neuromuscular physician with no family history of hereditary neuromuscular disease who presented with prolonged, episodic muscle rigidity causing significant functional limitations. Next-generation sequencing identified a heterozygous calpain 3 (CAPN3) variant [*NM_000070.3(CAPN3):c.2393C > A (p.Ala798Glu)*] categorized as pathogenic for autosomal-recessive CAPN3-related limb girdle muscular dystrophy type 1 (LGMD R1), which is of unclear significance. He was treated with dantrolene and showed marked functional gains that were lost with attempts to wean off medication. This case suggests that there may be a subset of patients suffering from muscle tightness who benefit from dantrolene.

## 1. Introduction

Muscle stiffness or rigidity is commonly encountered in clinical practice and is a major cause of functional decline, particularly in older adults [1–3]. It reflects an increase in flexor and extensor muscle tone that produces a resistive torque across a joint's entire range of motion. This contrasts with spasticity, in which velocity-dependent activation of muscle spindle afferents results in hyperreflexia and increased resistance to rapid stretching of the muscle (clasp-knife phenomenon).

Whereas spasticity reflects increased excitability of lower motor neurons secondary to upper motor neuron lesions [4], rigidity may originate from central mechanisms (e.g., dystonia) or peripheral mechanisms, including the muscle fiber itself. Contractions originating from the muscle fiber may be electrically active (i.e., action potential–dependent myotonia) or electrically silent (i.e., action potential–independent myotonia) on EMG [5]. Electrically silent muscle contraction may be seen in myopathies, malignant hyperthermia, neuroleptic malignant syndrome, Brody syndrome, rippling muscle disease, and hypothyroidism (myoedema). Recent evidence suggests that increased muscle stiffness or rigidity is common in older adults, requiring more effort to produce the same movement and reducing range of motion around a joint [2].

We are unaware of any systematic investigation into the incidence or prevalence of muscle stiffness in adults, despite its known association with functional decline [1–3]. When encountered, the workup is often negative, which can be frustrating for patient and the provider. A negative workup leaves few options for pharmacotherapy, and the patients are provided only with range of motion exercises and physical therapy. Here, we describe a patient who presented with this common scenario of functionally limiting muscle stiffness and rigidity. This patient showed marked improvement on dantrolene.

## 2. Case Report

A 62-year-old neuromuscular physician with no significant past medical history presented for evaluation of prolonged, episodic muscle rigidity resulting in significant functional limitations. The first episode occurred at age 52 following a severe viral illness and lasted 6 to 8 months before resolving spontaneously. The second episode began two months prior to presentation following bilateral inguinal hernia surgery. No neuromuscular blocking agents or inhaled anesthetics were administered during the surgery.

During both episodes, the patient experienced continuous and painless muscle contraction in the proximal legs and pelvic girdle including the vastus lateralis, iliotibial band, and hamstrings. During each of the two episodes, co-contraction of antagonist muscles caused marked gait impairment. High-intensity motor tasks, such as playing pickleball (which he previously did avidly), were no longer possible. He required use of a hand rail for stairs and during each of the two episodes strained the short head of the biceps femoris attempting to walk downstairs unassisted. During the second episode, he strained the right sartorius muscle while rolling over in bed. Upper extremity muscles were unaffected.

Examination was normal except for increased tone in the hip girdle bilaterally. The tensor fascia lata was particularly affected causing the iliotibial band to be visibly tensed below the skin. Difficulty with hip abduction during swing phase resulted in lateral tip of the torso toward the planted leg creating a unique, waddling gait pattern with normal base and absent Trendelenburg sign.

Lower extremity nerve conduction and EMG studies performed while the patient was symptomatic were normal. Concentric needle EMG of the left tibialis anterior, gastrocnemius (medial head), vastus medialis, and tensor fascia lata showed no abnormal spontaneous activity and normal motor unit potentials with normal recruitment patterns. Visibly contracted muscles were electrically silent without myokymic, myotonic, neuromyotonic, or cramp discharges.

Laboratory evaluation showed normal electrolytes, TSH, CPK, and aldolase. Myositis autoantibodies were negative. Next-generation sequencing identified a heterozygous pathogenic calpain 3 (CAPN3) variant [*NM_000070.3(CAPN3):c.2393C > A (p.Ala798Glu)*], which is responsible for autosomal-recessive CAPN3-related LGMD R1. Pathogenicity of this variant is supported by ACMG criteria PM3, PS3, PM2, PP3, and PP2. However, the clinical significance of this finding in a heterozygous state is uncertain.

The patient began a trial of dantrolene. Within a week of titrating his dose to 50 mg three times daily (TID), he demonstrated significant symptomatic relief with reduced sensation of muscle tightness. On further titration to 100 mg TID, marked functional improvements occurred. His gait pattern normalized. He was able to ambulate stairs unassisted, and he was able to return to playing pickleball. There were no adverse effects from the dantrolene. The patient had no noticeable weakness at this dose and did not have any objective findings of weakness on examination. Because dantrolene carries a black box warning for hepatotoxicity, which may be life-threatening, liver function was carefully monitored prior to starting dantrolene and at 6-month intervals after dose titration. No abnormalities were detected. Three separate attempts to taper off dantrolene over the course of a year resulted in worsening of muscle tightness, difficulty running, and difficulty walking downstairs. Functional improvements again occurred with resumption of 100 mg TID dosing. After 1 year of the treatment, the patient was able to slowly taper off the medication without noticeable worsening of symptoms.

## 3. Discussion

The patient described in the case was found to have electrically silent muscle contraction that was responsive to dantrolene. While the cause of the patient's muscle symptoms remains unknown, the therapeutic effect of dantrolene may provide mechanistic insight.

Dantrolene acts directly on the skeletal muscle to inhibit sarcoplasmic reticulum (SR) calcium (Ca) release, reducing the rise in sarcoplasmic Ca levels following an action potential. Dantrolene is an effective muscle relaxant for chronic spasticity due to upper motor neuron dysfunction, in which increased persistent inward currents in spinal motoneurons leads to motoneuron hyperexcitability, hyperreflexia, and muscle spasms [4]. In this setting, dantrolene reduces the downstream effect of excessive muscle contraction by depressing excitation and contraction coupling.

Dantrolene is also effective in treating malignant hyperthermia, neuroleptic malignant syndrome, and Brody disease [6, 7], in which excessive muscle contraction occurs in the absence of motoneuron or muscle fiber hyperexcitability. In these diseases, aberrant SR Ca release in the absence of muscle fiber action potentials causes electrically silent muscle contraction, presenting clinically as increased stiffness or rigidity, which is responsive to dantrolene.

The patient's response to dantrolene supports a working hypothesis that his symptoms reflect a reversible rise in sarcoplasmic Ca within electrically silent muscle. Muscle stiffness/rigidity increases in frequency and severity with aging [2], and rodent studies suggest that oxidative modification of the skeletal muscle ryanodine receptor (RyR) promotes Ca leak from the SR in aged muscle [8]. Because dantrolene inhibits RyR-mediated Ca release, it would be expected to lower sarcoplasmic Ca levels and reduce rigidity. Whether the CAPN3 variant identified on next-generation sequencing contributes to this pathophysiology is unclear. Dysregulated Ca handling linked to RyR dysfunction is reported in several muscular dystrophies, including Duchennes [9], dysferlin-related LGMD R2 [10], β-sarcoglycan-related LGMD R4 [11], and calpain 3-related LGMD R1 [12]. Moreover, both CAPN3 and dysferlin are closely associated with the Cav1.1–RyR complex [10, 13], and in dysferlinopathy models, the Ca leak can be attenuated by dantrolene [10]. However, CAPN3-associated LGMD R1 is autosomal recessive, and heterozygous carriers are typically unaffected. Thus, any causal link remains speculative.

In conclusion, dantrolene may represent a potential therapeutic option for a subset of patients with muscle stiffness or rigidity despite an otherwise normal neurologic examination and workup. The observed response here supports the hypothesis of pathologic SR Ca release, while successful weaning suggests that the associated Ca elevation, though prolonged, is reversible and may be triggered by a systemic stressor. Nonetheless, definitive mechanistic conclusions cannot yet be drawn without further investigation.

## Data Availability

The data that support the findings of this study are available on request from the corresponding author. The data are not publicly available due to privacy or ethical restrictions.

## Nomenclature

Ca: Calcium
LGMD: Limb girdle muscular dystrophy
SR: Sarcoplasmic reticulum
TID: Three times daily
RYR: Ryanodine receptor
